# Oncological and functional outcomes of focal irreversible electroporation for localised prostate cancer: a single institution series of 171 cases

**DOI:** 10.1038/s41391-026-01148-x

**Published:** 2026-09-01

**Authors:** Z. Mian, T. Gianduzzo, B. Kua, J. Yaxley

**Affiliations:** 1 School of Medicine, Bond University, Gold Coast, QLD Australia; 2 Wesley Hospital, Brisbane, QLD Australia; 3 Department of Medicine, University of Queensland, Brisbane, QLD Australia; 4 Department of Urology, Royal Brisbane and Women’s Hospital, Brisbane, QLD Australia

**Keywords:** Prostate cancer, Cancer therapy

## Abstract

**Background:**

Focal irreversible electroporation (IRE) is an attractive choice for patients aiming to obtain local control of prostate cancer, while decreasing the postoperative risk of impotence and incontinence compared to whole-gland treatments.

**Methods:**

Retrospective review of a single centre prospective database of 171 consecutive primary IRE procedures for localised prostate cancer. Primary outcomes include oncological infield and out-of-field progression. Secondary objectives include post-IRE continence and impotence.

**Results:**

The median follow-up is 35 months (3–90 months). The median diameter of the treated lesions is 11 mm (4–24 mm). Most patients had PI-RADS 4–5 abnormalities on prostate MRI, and 25 patients (15%) had ISUP grade 4–5 cancer. Of the 135 patients with >12 months follow-up, 84 had a surveillance biopsy of whom 8.3% had significant infield persistence, with significant out-of-field cancer in 14.3%. There was no difference in the risk of infield cancer persistence regardless of the pre-IRE ISUP score (*p* = 0.8), or tumour location (*p* > 0.9). The presence of low volume out-of-field cancer at diagnosis did not increase the risk of post-IRE cancer persistence/progression (*p* = 0.7). The prostate PSMA PET SUVmax of ≥10 did not increase the risk of infield or out-of-field recurrence of cancer (*p* = 0.3). A PSAD > 0.15 did not increase the risk of infield persistence or out-of-field progression of cancer (*p* = 0.8). Urinary continence was maintained in 167/171 (97.6%), while erectile function was maintained in 94/112 (84%) of sexually active patients who completed questionnaires.

**Conclusions:**

Of the patients who proceeded to post-IRE biopsy, 92% had infield ablation of clinically significant cancer with a low risk of urinary incontinence or impotence. Focal IRE of prostate cancer has promising short to medium-term outcomes and is associated with low risks of impotence and incontinence. Long-term follow-up of out-of-field progression risk is required.

## Introduction

Whole gland treatment of localised prostate cancer with a radical prostatectomy or radiotherapy techniques as a primary or multimodality combination, offers excellent long-term clinical local control of prostate cancer. However, focal local ablation of prostate cancer is becoming a more popular treatment as patient’s seek a potential cure, while reducing the known side effects of whole-gland treatment such as urinary incontinence, erectile dysfunction, and radiation-related changes to the bowel and bladder. Clinician concern regarding focal therapy includes the need for evidence of histological infield ablation for local control, and the potential long-term risk of progression of cancer outside the focal ablation zone.

Focal irreversible electroporation (IRE) applies an electric field to targeted cells, increasing cell membrane permeability and inducing the formation of irreversible nanopores within the cell membrane [1]. Cell death after focal IRE occurs by apoptosis or necrosis, with the potential for preservation of blood vessels, endothelial and smooth muscle cells. This results in nonthermal ablation, potentially decreasing the risk of damage to surrounding tissue such as urethra, external sphincter, bladder neck, neurovascular bundle and rectum.

Patient selection for focal therapy is evolving. Transperineal template biopsy procedures and multiparametric MRI improve the identification and evaluation of target lesions. Prostate-specific membrane antigen positron emission tomography (PSMA-PET) imaging may further aid in the identification of non-MRI-visible disease [2]. A recent meta-analysis of focal IRE identified significant variations in the published risk of infield persistence of prostate cancer (0–33%) and out-of-field progression (0–37%), with median follow-ups ranging from 6 to 16 months [3]. The meta-analysis demonstrated the potential quality-of-life benefits of focal IRE, with post-IRE preservation of baseline erections suitable for intercourse reported in 78–97% of patients and urinary incontinence rates of 1–2%.

The aim of our study was to document our single centre oncological and functional outcomes following unilateral focal IRE and evaluate the need for secondary salvage treatments for infield persistence of cancer or out-of-field progression. We also aimed to evaluate if histological low-grade or low-volume intermediate-risk prostate cancer on template/systematic biopsies outside the focal IRE treatment zone on the initial diagnostic biopsy increases the short-medium term risk of out-of-field progression requiring secondary treatments. Other secondary aims included whether the prostate PSMA PET SUVmax and PSA density influenced infield persistence or out-of-field progression of clinically significant prostate cancer (csPC).

## Methods

This is a single-centre retrospective review of three urological surgeon’s prospective database, approved by the Uniting Care Health Human Research Ethics committee, reference: 2025.27.435. All methods were performed in accordance with the relevant guidelines and regulations. The study was performed in accordance with the Declaration of Helsinki. Consecutive patients were included from our first IRE ablation in August 2018 up to November 2025. A multiparametric prostate MRI was performed prior to the prostate biopsies, which included target and template biopsies. Localised cancer was confirmed by staging with a PSMA PET scan for intermediate and high-risk prostate cancer.

Inclusion criteria included patients aged >50 years, with only unilateral prostate ablations included for analysis. Patients with seminal vesicle invasion, radiological evidence of extraprostatic extension, salvage IRE for post radiation therapy local recurrence were excluded. Anterior tumours included the transitional zone (TZ), anterior fibromuscular area or anterior horn of peripheral zone (PZ) above the level of the urethra. If a tumour crossed both zones it was classified as a PZ malignancy. There was no exclusion based on PSA levels, ISUP grade or PSA density.

All prostate biopsies were via a transperineal approach including template sampling with the number of cores determined at the surgeon’s discretion (median 21 cores; range 8–48 cores). The treatment zone was based on the PI-RADS 3–5 lesion on MRI, or if the MRI scan was negative (PI-RADS 1–2), based on the anatomical location of the positive histology if concordant with a PSMA-avid prostate focus on the PET scan. The treatment zone included a minimal 5 mm margin around the radiological region of interest. Patients without a visible radiological lesion were not treated with focal IRE.

The IRE procedure was performed under general anaesthesia with using the NanoKnife (Angiodynamics PTL, Latham, NY, USA) computer-based treatment planning software machine. The current delivered between each IRE needle ranged from a minimum of 25 A to maximum 50 A. The IRE needle exposure length ranged from 10 to 25 mm and the distance between each needle ranged from 8 to 23 mm. A continuous run of 80–90 pulses was delivered between each IRE needle. The timing of the indwelling catheter removal was dependent on the individual surgeon.

Surveillance prostate biopsies were usually performed at 12–24 months post treatment at the surgeon’s discretion. Significant tumour recurrence was defined as a ≥6 mm core of International Society of Urological Pathology (ISUP) grade 1 (Gleason score 3 + 3), or ≥4 mm tumour length ISUP grade 2 (Gleason 3 + 4), or any focus of ISUP grade ≥3 (Gleason ≥4 + 3). Lesions with a positive core length of <4 mm are associated with tumour volume of <0.2 cc on whole mount histology [4].

Erectile function was defined as moderate (or greater) confidence in the ability to obtain and keep an erection on the International Index of Erectile Function (IIEF-5) score, or as small or no problem obtaining an erection based on the Expanded Prostate Cancer Index Composite (EPIC) questionnaire. Urinary incontinence was defined as the requirement for any pad use following the IRE procedure.

Statistical analyses were performed using R (version 4.4.0) with the R packages tidyverse and gtsummary. Wilcoxon rank sum test was used to compare continuous variables between groups. Fisher’s exact test was used to compare the frequency of categorical variables (e.g. recurrence rates) between groups. To account for potential confounding, multivariable logistic regression to predict recurrence was performed on clinical variables of interest.

The primary oncological endpoint was in-field csPCa on biopsy performed within 12–24 months after IRE, or later depending on patient consent. Secondary oncological endpoints included out-of-field csPCa; time to salvage treatment, recurrence-free survival, influence of SUVmax and PSA density on infield persistence or out-of-field progression of csPC, and functional outcomes including pad-free continence and sexual function.

Intraoperative events and postoperative adverse events were classified as per the Clavien–Dindo classification.

## Results

The demographics are listed in Table 1. The median follow-up of the entire cohort is 35 months (3–90 months). There were 171 primary IRE patients eligible for inclusion. The median PSA was 5.4 μg/L (0.8–25 μg/L). The prostate volumes ranged from 19–105 cc with a median PSA density (PSAD) of 0.12 ng/mL/cc. The median length of stay was one day (0–2 days). The median diameter of the treated lesions was 11 mm (4–24 mm).

**Table 1:** Clinical characteristics of cohort, *n* = 171.

Cohort demographics	Biopsy done *N* = 84^a^	No biopsy done *N* = 87^a^	*p*-value^b^
Age at IRE (years)	70 (51, 87)	70 (53, 87)	>0.9
Unknown	0	1	
PSA at diagnosis (ug/L)	5.8 (0.8, 16.0)	4.9 (1.3, 25.0)	0.075
Length of stay (days)			0.11
0	8 (9.5%)	2 (2.3%)	
1	73 (87%)	83 (95%)	
2	3 (3.6%)	2 (2.3%)	
PSA density	0.13 (0.01, 1.00)	0.17 (0.04, 1.00)	0.11
Unknown	1	2	
Prostate volume (CC)	47 (15, 105)	41 (22, 92)	0.007
Not reported	1	2	
Diameter L1 (mm)	11.0 (0.0, 22.0)	11.0 (2.2, 24.0)	0.4
Not reported	7	20	
Follow up (months)	55 (15, 90)	15 (3, 90)	<0.001
PIRADS on MRI			0.033
2	3 (3.6%)	14 (16%)	
3	6 (7.1%)	9 (10%)	
4	63 (75%)	53 (61%)	
5	11 (13%)	11 (13%)	
not reported	1 (1.2%)	0 (0%)	
Highest ISUP			0.2
1	3 (3.6%)	6 (6.9%)	
2	35 (42%)	48 (55%)	
3	30 (36%)	24 (28%)	
4	8 (9.5%)	4 (4.6%)	
5	8 (9.5%)	5 (5.7%)	

^a^Median (Min, Max); *n* (%).

^b^Wilcoxon rank test; Fisher’s exact test.

Most patients had PI-RADS 4–5 abnormalities on the prostate MRI. There were 62 anterior/transitional zone lesions (TZ), and 25 patients (15%) had high-risk ISUP grade 4–5 cancer.

### Oncological outcomes

The prostate cancer-specific survival is 100% and overall survival is 98.8% (169/171). In total, 21 of the 135 (16%) patients with more than 12 months follow-up have proceeded to salvage local treatment; 15 proceeded with a robot-assisted laparoscopic radical prostatectomy (RALP), four with image-guided radiotherapy, and two with brachytherapy. Three other patients with metastases were treated with androgen deprivation therapy and systemic agents. The outcomes of the post-IRE surveillance biopsies are shown in Table 2. Surveillance post-IRE prostate biopsies were performed in 40 of the first 50 patients (80%) treated, decreasing to 84/135 (62.2%) of the patients with more than 12 months follow-up. As shown in Table 3, the surveillance no-biopsy group had shorter follow-up, smaller prostate volumes and lower PIRADS scores. Of the 51 patients who did not have a follow-up biopsy, 49/51 have no PSA progression, including 39/51 with a >50% reduction in pre-IRE PSA. One patient was commenced on ADT for supraclavicular metastasis on follow-up PSMA PET for PSA progression. In this group without follow-up biopsy, 37/51 also had a surveillance MRI beyond 12 months of which 36/37 were PIRADS ≤3 and one small PIRADS 4 lesion, which is stable under surveillance. Another had a negative surveillance PSMA PET.

**Table 2:** Outcomes of post-IRE prostate biopsies.

Characteristic	Out-of-field lesion present at diagnosis *N* = 38^a^	No out-of-field lesion at diagnosis *N* = 133^a^
12 months post-IRE biopsy results		
Significant in-field recurrence	1 (2.6%)	6 (4.5%)
Significant out-of-field recurrence	4 (11%)	8 (6.0%)
Insignificant in-field recurrence	1 (2.6%)	4 (3.0%)
Insignificant out-of-field recurrence	6 (16%)	16 (12%)
Clear	9 (24%)	29 (22%)
No biopsy done	17 (45%)	70 (53%)

^a^*n* (%).

**Table 3:** PSA and MRI characteristics of patients with >12 months follow-up.

Characteristic	Biopsy done *N* = 84^a^	No biopsy done *N* = 51^a^	*p*-value^b^
Follow up (months)	55 (15, 90)	35 (13, 90)	0.018
PSA at diagnosis (ug/L)	5.8 (0.8, 16.0)	5.0 (1.3, 25.0)	0.2
PSA density	0.13 (0.01, 1.00)	0.17 (0.04, 1.00)	0.12
Unknown	1	2	
Prostate volume (CC)	47 (15, 105)	36 (22, 77)	0.002
Not reported	1	2	
Diameter L1 (mm)	11.0 (0.0, 22.0)	11.0 (2.2, 22.0)	0.7
Not reported	7	13	
PIRADS on MRI			0.007
2	3 (3.6%)	11 (22%)	
3	6 (7.1%)	6 (12%)	
4	63 (75%)	28 (55%)	
5	11 (13%)	6 (12%)	
Not reported	1 (1.2%)	0 (0%)	
Highest ISUP			0.8
1	3 (3.6%)	3 (5.9%)	
2	35 (42%)	26 (51%)	
3	30 (36%)	15 (29%)	
4	8 (9.5%)	3 (5.9%)	
5	8 (9.5%)	4 (7.8%)	

^a^Median (Min, Max); *n* (%).

^b^Wilcoxon rank sum test; Fisher’s exact test.

### Infield recurrence

Significant infield cancer persistence occurred in 7/84 (8.3%) patients, including three patients with ISUP grade 2, three with ISUP 3 and one with ISUP 4–5 malignancies (Table 4). There was no difference between the initial pre-IRE histological ISUP score and the risk of infield cancer persistence (*p* = 0.8), or anterior/TZ or peripheral zone (PZ) ablations (*p* > 0.9). Insignificant infield persistence was identified in 5/84 (6%), including one patient with low volume <4 mm ISUP grade 2. There was also no statistical difference on multivariable analysis, but the low recurrence rates prevented a meaningful multivariate analysis.

**Table 4:** In-field cancer recurrence by Initial ISUP grading.

Initial ISUP grade	Number of cases	Number of post-IRE biopsies	Insignificant in-field recurrence	Significant in-field recurrence
1	9	3	0	0
2	83	35	2	3
3	55	30	3	3
4	11	8	0	1
5	13	8	0	0

### Out-of-field recurrence

Significant out-of-field cancer was identified in 12/84 (14.3%) patients. There was no difference in significant out-of-field progression regardless of whether the IRE ablation was performed in the PZ or TZ (*p* = 0.7). Prior to primary IRE, 38/171 (22.2%) had histological evidence of low volume out-of-field prostate cancer, (ISUP 1 in 26, ISUP 2 in nine and a focus of ISUP 3–4 in three patients. Of these 28/38 have more than 12 months follow-up, with 21/28 (75%) proceeding to surveillance biopsies. Four of these patients (4/21, 19%) have developed histological csPC out-of-field progression, and one significant infield progression. The remaining 16 had either no cancer on repeat biopsy or insignificant malignancy suitable for surveillance, including two with low volume <4 mm ISUP 2. Of the remaining seven non-biopsy patients all remain on surveillance, with low-risk follow-up MRI scans and stable or slowly rising PSA levels.

Of the 133 patients with no out-of-field malignancy at the time of focal IRE, 63 proceeded to a surveillance biopsy. Of these 63 patients, 29 (46%) had negative biopsies, 16 (25%) insignificant out-of-field biopsies (of which eight were low volume <4 mm ISUP 2) and eight (13%) developed significant out-of-field progression. Four patients had significant infield disease and six insignificant infield persistence of cancer. There were 70 patients who did not proceed to surveillance biopsy based on low-risk clinical/radiological parameters or they were not due for a biopsy on follow-up protocols.

The presence of low volume out-of-field cancer at diagnosis did not increase the risk of cancer persistence/progression (*p* = 0.7) compared to no out-of-field cancer at diagnosis, including significant out-of-field progression (*p* = 0.5).

### Prostate SUVmax at diagnosis and PSA density

The prostate SUVmax was recorded in 135 of the pre-IRE PSMA PET scan reports, with 32/135 (23.7%) having a SUVmax of ≥10. Of the 65 patients with SUVmax recorded that proceeded with a surveillance biopsy, the presence of an index lesion SUVmax of 10 or higher did not increase the risk of either infield persistence or out-of-field recurrence of cancer (*p* = 0.3), as outlined in Table 5, including significant infield persistence of cancer (*p* = 0.6).

**Table 5:** Recurrence by SUV max - No biopsy done is not displayed.

	SUV max		
Characteristic	<10 *N* = 51^a^	10 or higher *N* = 14^a^	*p*-value^b^
Biopsy results			0.3
Clear	23 (45%)	4 (29%)	
Insignificant out-of-field recurrence	14 (27%)	5 (36%)	
Insignificant in-field recurrence	2 (3.9%)	2 (14%)	
Significant out-of-field recurrence	7 (14%)	3 (21%)	
Significant in-field recurrence	5 (9.8%)	0 (0%)	

^a^*n* (%).

^b^Fisher’s exact test.

The PSA density (PSAD) was available in 168/171 patients with 52% having a PSAD of <0.15 ng/mL. A PSAD >0.15 did not increase the risk of infield persistence or any out-of-field progression of cancer (*p* = 0.8).

### Quality of life continence and sexual outcomes

Of the 114 sexually active patients prior to the focal IRE, erectile function suitable for satisfactory intercourse with or without the use of an oral phosphodiesterase inhibitor was maintained in 94/112 (84%) of patients who completed the follow-up questionnaires. There was no difference between recovery of sexual function between PZ or anterior/TZ ablations (86% vs 81%, *p* = 0.6).

Urinary continence defined as no pad use was maintained in 167/171 (97.6%), prior to salvage treatments. A history of prior transurethral resection of the prostate was identified in 14 patients, none of whom developed new incontinence following the IRE procedure. None of the four patients who required a TURP either at the time of IRE or postoperatively developed urinary incontinence.

### Complications

Clavien ≥3 complications within three months post-IRE occurred in five patients, with three requiring a TURP for persistent retention and two patients required dilation of a urethral stricture. In this series, 12 of the 15 salvage RALP procedures were free of a pad at last follow-up, with only one requiring more than one pad per day.

## Discussion

Our results indicate that focal IRE provides good histological ablation in the treatment zone. The decision to proceed with a post-IRE biopsy remained a decision between the individual surgeon and patient. Not all patients with over 12 months follow-up agreed to a surveillance biopsy, usually due to low post-IRE PSA levels associated with low-risk follow-up MRI results. Only 8.3% of our patients have infield persistence of clinically significant prostate cancer and 85.7% had eradication of all malignant cells. Over time individual surgeons have become comfortable with increasing the ablation zone margin where technically possible, noting the lack of functional difference between focal and hemi-gland ablation after six months follow-up in a randomised control trial [5].

We used a definition of >6 mm of ISUP grade 1 or ≥4 mm of ISUP grade 2 as significant prostate cancer based on whole mount histology at radical prostatectomy [4]. The histological definition of a significant prostate cancer is not always concordant with clinical decisions on management of low volume ISUIP grade 2-3 malignancy. The use of the histological “clinically significant” malignancy can influence both treatment pathways and clinical outcomes and may lead to unnecessary interventions or delayed treatment, affecting patient prognosis and healthcare resources [6]. Further, a proportion of men with “clinically significant” cancer will never experience clinical symptoms, even if untreated [6].

In the Zhang et al. prospective observational multi-centre study [7], 24% of patients had clinically significant infield or out-of-field ISUP grade ≥2 on the post-IRE biopsy at 12–18 months. This compares to 22.6% of our cohort, combining both infield and out-of-field cancer. However, in our cohort 12/171 (7%) already had a focus of ≥ISUP 2 out-of-field cancer prior to focal IRE. The IRE target zone was clear of any cancer in 85.7% of our patients, which compares favourably to the PRESERVE trial [8] where excluding patients without 12-month biopsy data, the rate of negative in-field biopsies was 80% (86/107). It is unknown why prostate cancer persists in some patients following focal IRE. This is most likely a multifactorial process due to inadequate margin status of the ablation zone, or the IRE needle placement position and/or arrangement around the ablation zone resulting in small areas of suboptimal exposure to the IRE current. Alternatively, more than a continuous run of 80–90 pulses between each IRE needle or a higher minimal delivered current is required to cause irreversible damage to the cell membrane. This is an area for further research.

Focal therapy has usually only been recommended for intermediate risk or high-volume low-grade prostate cancer [9]. However, 15% of our cohort had high-grade prostate cancer and we did not find any difference in the probability of histological infield persistence compared to the intermediate risk or low-grade cancers. There is an increased risk of biochemical recurrence following any whole treatment of ISUP 4–5 prostate cancer, most likely due to a higher probability of microscopic extraprostatic extension or micrometastatic disease not identified on staging radiology. We are currently completing a multicentre review of the outcomes of focal IRE for high-grade prostate cancer. It is well known that a higher SUVmax on a PSMA PET scan is associated with a higher-grade prostate cancer on biopsy. However, we did not find that a high SUVmax >10 altered the infield ablation of prostate cancer.

Of the patients with more than 12 months of follow-up, only 16.2% have proceeded with salvage local treatments. We have not treated local recurrence with repeat focal ablation. Although not the subject of this study, the authors can confirm there was no increased intraoperative or postoperative complications with the salvage RALP procedures compared to primary surgical treatment of prostate cancer. A very important finding is that the presence of low-volume out-of-field cancer at diagnosis did not increase the risk of cancer persistence or progression compared to no out-of-field cancer at diagnosis, including significant out-of-field progression. Therefore, out-of-field low-volume disease should not be an absolute contraindication for patients who wish to maximise short-medium term quality of life outcomes with the understanding that long-term outcomes are still unknown.

There remains clinician concern regarding the lack of long-term oncological outcomes following focal therapy. In patients with low- intermediate risk prostate cancer, oncological outcomes over 8 years were similar between focal therapy using HIFU or cryotherapy compared to a radical prostatectomy [10]. In the Scheltema focal IRE series of 229 patients with a median follow-up of 60 months, the Kaplan–Meier failure free survival rate was 69% at 8 years, with 17% of patients proceeding to radical salvage treatments [11]. For our patients with a minimal follow-up of 5 years, 10/45 (22.2%) have required salvage treatment. Importantly, focal IRE does not exclude salvage whole-gland therapy for local recurrence.

The suitability to perform focal therapy in the presence of a contralateral low volume out-of-field histological malignancy on the template biopsies is becoming less controversial in contemporary urology. Most focal therapy programs do not to exclude patients with low volume out-of-field prostate cancer, as these patients are suitable for active surveillance based on most modern guidelines. Longer-term outcomes are required but based on the active surveillance results of the ProTect trial [12], it is conceivable that a significant proportion of these patients will not require treatment for out-of-field progression over the following decade. The hypothesis is for focal IRE to remove the tumour most likely to metastasise, with the option of salvage therapy if the out-of-field prostate cancer progresses in the future. Leni et al. found a significant number of patients with low volume ISUP grade 2 on initial biopsy are downgraded to less aggressive ISUP grade 1 or no cancer at confirmatory biopsy [13]. Our results showed 46% of patients with out-of-field malignancy on initial template biopsies had negative out-of-field biopsies on surveillance, with only 19% developing histological clinically significant out-of-field progression during the study period.

A match-paired analysis demonstrated the superior preservation of urinary continence and erectile function with IRE compared to RALP [14]. Randomised controlled trials are currently underway to compare functional and oncological outcomes of focal IRE to radical treatment of prostate cancer, including the PIAS [15] and PART [16] trials. Based on the definition of no pad use, 97.6% of our patients maintain pad free urinary continence following focal IRE. These results are consistent with prior publications, however, in some IRE studies, as with many published series on radical prostatectomy outcomes, a definition of 0–1 pad per day has been defined as continence.

A patients’ quality of life and mental health can be significantly negatively impacted by incontinence of urine and impotence after prostate cancer treatment. Focal therapy offers an ontologically sound treatment option to minimise the risk of these post-treatment functional side effects. Our results show that 84% of the sexually active patients maintain erectile function suitable for satisfactory intercourse. In contrast, the favourable early continence and erectile function results must be balanced against the lack of long-term oncological outcomes beyond 10 years following focal IRE, including limited evidence regarding functional outcomes after salvage local treatments if required. Reassuringly, in the Blazevski et al. small series of post-IRE salvage robot-assisted laparoscopic radical prostatectomy (s-RALP) by a very experienced high-volume urological surgeon all achieved pad-free status by six months and the majority (60%) recovered erectile function [17]. Whether similar results can be achieved in the real world with varying surgeon experience and techniques will require further evaluation. The Marconi et al. multicentre cohort of 82 patients undergoing s-RALP after focal therapy failure reported continence rates of 83% using a no pad definition [18], with erections hard enough for penetration decreasing from 67% at baseline to 14% at 12 months.

As this was not a pilot study, a limitation includes surveillance prostate biopsies were not mandated, but instead performed on clinician and patient interpretation of risk. Patient preference to avoid the follow-up biopsy was due to low PSA +/− a low risk surveillance MRI results, or in one patient metastatic progressive disease on surveillance radiology. Routine post-IRE 12-month prostate biopsies have decreased over the period of our IRE program as clinicians become comfortable with the high infield clearance of clinically significant cancer. However, research has shown that the combination of PSA and MRI alone are not sufficient to completely omit surveillance biopsies during follow-up [19, 20]. Part of our ongoing research is whether the addition of a pre- and post-IRE surveillance PSMA PET scan can better predict a patient’s risk of post-IRE recurrence. Further, the infield persistence rate of 8.3% applies only to the biopsied subset, which had longer follow-up and higher-risk features. Therefore, this figure likely represents an upper level for the true infield persistence rate in the entire cohort.

Although focal primary IRE was associated with a 92% rate of in-field ablation of clinically significant cancer among patients in our cohort who underwent post-IRE biopsy, the long-term risk of out-of-field progression requires further investigation. Additionally, long-term prostate cancer-specific survival outcomes comparing early salvage treatment for local recurrence after focal therapy with upfront whole-gland treatment remain uncertain. Our early results indicate that low volume favourable out-of-field contralateral cancer does not increase the risk of requiring salvage local treatments over a median follow-up of 35 months. Unfortunately, long-term oncological results of randomised trials between focal and whole-gland treatment will not be known for some time. It is therefore important for clinicians continue to research focal therapy outcomes and define which patients are suitable for focal therapy.

## Data Availability

The datasets generated and analysed during the current study are available from the corresponding author on reasonable request, subject to local ethics approval.
